# Lung microbiome signatures and explainable predictive modeling of glucocorticoid response in severe community acquired pneumonia

**DOI:** 10.3389/fmicb.2025.1706432

**Published:** 2025-11-28

**Authors:** Yeong-Nan Cheng, Guan-Ting Chen, Wei-Chih Huang, Yen-Peng Chiu, Yun Tang, Pin-Kuei Fu, Tzong-Yi Lee

**Affiliations:** 1 Institute of Bioinformatics and Systems Biology, College of Engineering Bioscience, National Yang Ming Chiao Tung University, Hsinchu, Taiwan; 2 Department of Biological Science and Technology, College of Engineering Bioscience, National Yang Ming Chiao Tung University, Hsinchu, Taiwan; 3 Institute of Data Science and Engineering, College of Computer Science, National Yang Ming Chiao Tung University, Hsinchu, Taiwan; 4 Department of Medical Research, Taichung Veterans General Hospital, Taichung, Taiwan; 5 Department of Post-Baccalaureate Medicine, College of Medicine, National Chung Hsing University, Taichung, Taiwan; 6 Center for Intelligent Drug Systems and Smart Bio-devices (IDS2B), National Yang Ming Chiao Tung University, Hsinchu, Taiwan

**Keywords:** severe community-acquired pneumonia (SCAP), systemic glucocorticoids response, gut-lung axis, machine learning, lung microbiome

## Abstract

**Introduction:**

Systemic glucocorticoids (SG) are administered to quell hyper-inflammation in severe community acquired pneumonia (SCAP), yet trials report inconsistent efficacy and no mechanistic explanation.

**Methods:**

We enrolled 200 ventilated SCAP patients, whom received hydrocortisone within 48 h of ICU admission, and generated longitudinal lower-airway microbiome profiles by 16S rRNA amplicon and metagenomic sequencing on ICU Days 1, 3 and 7. Compositional data were integrated with clinical variables through a fully reproducible bioinformatics analysis workflow.

**Results:**

Baseline community structures did not differ between SG and control cohorts, but by Day 7 survivors exhibited enrichment of Actinobacteria and Gammaproteobacteria whereas non-survivors accumulated Alphaproteobacteria and Campylobacteria. A random-forest model restricted to Bacilli and Alphaproteobacteria achieved AUROC = 0.89 (sensitivity 0.83, specificity 0.81) on a patient-held-out test set, significantly outperforming conventional severity indices like APACHE II, SOFA and mNUTRIC scores.

**Discussion:**

Collectively, our results demonstrate that SG therapy imposes reproducible ecological pressures on the lung microbiome and that a two-feature microbial fingerprint can forecast treatment success with single-sample resolution. These findings show that SG therapy actively reshapes the respiratory ecosystem and that lightweight microbiome-aware machine learning can stratify treatment response, offering a tractable path toward precision corticosteroid stewardship.

## Background

Community-acquired pneumonia (CAP) is a leading cause of morbidity and mortality, often requiring intensive care unit (ICU) admission due to complications such as septic shock and acute respiratory distress syndrome (ARDS) (Meduri et al., 2022). Severe CAP (SCAP) is a critical form of the disease with a high mortality risk (Lin et al., 2023; Nair and Niederman, 2021; Saxena et al., 2020). *Streptococcus pneumoniae* is the most frequently isolated bacterial pathogen (Niederman and Torres, 2022), while excessive inflammation exacerbates disease severity, leading to treatment failure and increased mortality. To counteract inflammation, systemic glucocorticoids (SG) have been proposed for their immunosuppressive effects (Iqbal et al., 2020). A recent clinical trial demonstrated that SG significantly reduced the 28-day mortality rate in SCAP patients (Dequin et al., 2023). However, other studies report mixed results, with some showing benefits such as faster clinical stabilization (Dequin et al., 2023; Wu et al., 2023; Nora et al., 2020; Ricard and Messika, 2015), while others highlight risks, including prolonged hospitalization and no mortality reduction (Meduri et al., 2022; Iqbal et al., 2020; Saleem et al., 2023; Torres et al., 2015; File, 2015). Additionally, SG appears ineffective against pneumococcal pneumonia and may be harmful in influenza infections (Nora et al., 2020).

Advances in high-throughput sequencing, including metagenomic next-generation sequencing (mNGS), have improved microbial profiling and pathogen identification in respiratory infections (Lin et al., 2023; Fan et al., 2022; Jin et al., 2022). Specific microbial profiles have been shown to predict prognosis in patients with severe pneumonia (Huang et al., 2024; Han et al., 2025); however, the effects of SG treatment on the lung microbiome in SCAP remain unclear. Little is known about the impact of SG on the lung microbiome even in other respiratory diseases. In a study of acute exacerbations of chronic obstructive pulmonary disease (COPD) (Huang et al., 2014), treatment with SG alone was found to lead to increased diversity and predominant enrichment of Proteobacteria, indicating that it may be associated with increased bacterial burden and elevated abundance of specific microbiota. In a study of lower respiratory tract infections (Li et al., 2024a), associations were noted between SG treatment and specific microbiota, including *Leptotrichia buccalis, Pelodictyon luteolum* and *Staphylococcus phage StB12*. In a study of pneumonia-associated ARDS (Shen et al., 2022), Polyomaviridae, Anelloviridae, Herpesviridae and Pneumocystidaceae are more abundant in SG responders; for the non-responders, taxa of higher abundances were Xanthomonadaceae, Debaryomycetaceae, Moraxellaceae, Enterococcaceae and Pseudomonadaceae. On the other hand, inhaled corticosteroids (ICS) have been shown to influence the lung microbiome in conditions such as asthma, COPD and rhinitis (Hartmann et al., 2021; Toraldo and Conte, 2019; Toraldo et al., 2022), but it is not valid to extrapolate from the therapeutic outcomes and microbial-community shifts observed with ICS in other respiratory diseases to the expected response to SG in patients with SCAP.

The integration of artificial intelligence (AI) with metagenomics offers a promising approach to identifying complex microbial patterns, facilitating more precise diagnostics and treatment strategies. For instance, bioinformatics pipelines enhance respiratory pathogen detection in sequencing data (Viana et al., 2024), emphasizing the need for rapid and accurate analysis. This study analyzed mNGS data from ICU-admitted CAP patients and employed 16S rRNA sequencing to characterize SCAP microbial profiles. AI-based feature selection and model development were applied to assess the impact of SG treatment on lung microbiome composition. Identifying SCAP patients who would benefit from SG remains challenging due to the lack of definitive clinical markers. Machine learning (ML) offers a potential solution by uncovering predictive patterns in complex datasets (Ferreiro et al., 2023). We hypothesize that explainable ML analysis of lung microbiome profiles can reveal biomarkers predictive of SG responsiveness, offering a precision medicine approach where conventional clinical indicators are insufficient. By leveraging high-throughput sequencing, we aim to characterize the microbial composition in SCAP patients and determine the impact of SG treatment. Our objective is to identify microbial taxa that could serve as predictive markers for SG treatment efficacy in SCAP, thereby improving clinical decision-making.

## Materials and methods

### Study design and enrollment of patients

This cohort study was conducted in the ICU of Taichung Veterans General Hospital (TCVGH) from September 2017 to May 2018. It was approved by the TCVGH Institutional Review Board (IRB No. SE17214B) and registered on ClinicalTrials.gov (NCT03379779). Adult patients (>40 years) admitted with pneumonia-related sepsis, septic shock, or acute respiratory failure were included. Patients with SCAP requiring mechanical ventilation within 48 hours were enrolled, while those with active pulmonary tuberculosis or those who had received antibiotics for more than seven days within 2 weeks before pneumonia onset were excluded. Written informed consent was obtained from all participants.

**Figure 1:**
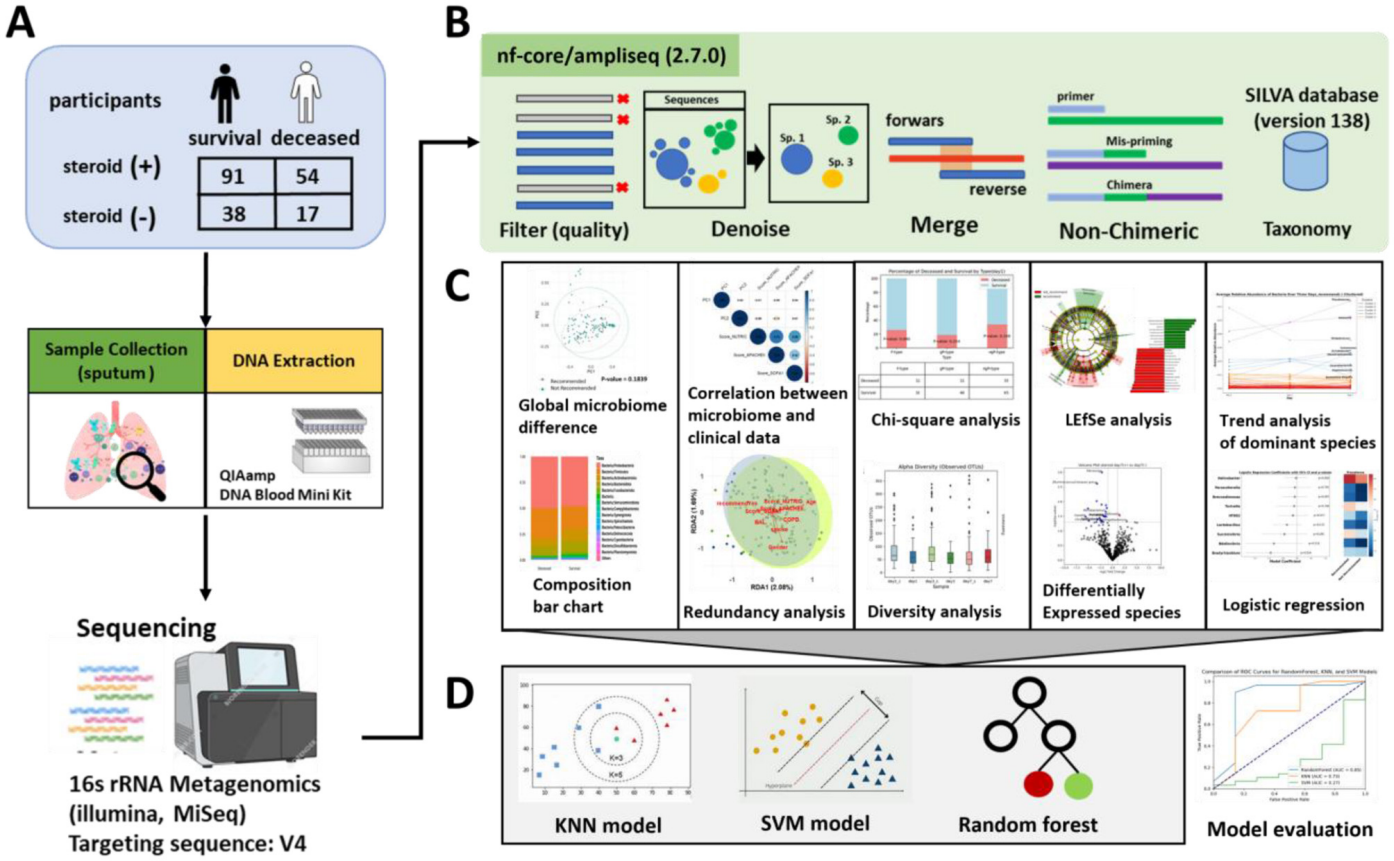
Research workflow diagram. **(A)** Overview of the study, including the collection of lung microbiome samples and sequencing. **(B)** Sequence data preprocessing and microbial species identification using nf-core/ampliseq (2.7.0). **(C)** Statistical analyses for microbiome data observation and feature selection. **(D)** Development and validation of a machine learning model to predict corticosteroid treatment recommendations.

Standard ICU management included low tidal volume ventilation for acute respiratory distress syndrome, nutritional support, antibiotic therapy, and hemodialysis as needed. SCAP patients with septic shock received intravenous hydrocortisone (200 mg daily for 7 days). Bronchoscopic examinations were performed by three full-time ICU physicians who were also pulmonologists. Nutritional status was assessed using the modified NUTRIC (mNUTRIC) score (Wang et al., 2019) by a registered dietitian, while disease severity was evaluated using the Acute Physiology and Chronic Health Evaluation (APACHE) II and Sequential Organ Failure Assessment (SOFA) scores (Singer et al., 2016; Taylor et al., 2016) (Figure 1).

### Data collection, microbial culture, identification, and DNA extraction

Data collected from case report forms included age, sex, body mass index (BMI), and clinical outcomes such as illness severity scores (APACHE II and SOFA scores on day 1, 3, and 7 post ICU admission), major comorbidities and the Charlson Comorbidity Index (CCI), number of ventilator days, total hospital and ICU length of stay, and mortality in the ICU or hospital. Lower respiratory tract specimens, including endotracheal aspirate (EA) collected routinely and bronchoalveolar lavage (BAL) collected selectively on the first day of ICU admission, were processed for both conventional microbiological analysis and metagenomic sequencing. Each EA was inoculated onto standard bacterial and fungal culture media and incubated for up to 7 days, after which culture results were compared with metagenomic next-generation sequencing (mNGS) data for comprehensive microbial profiling. In parallel, aliquots of EA and BAL samples were sent for DNA extraction using the QIAamp DNA Blood Mini Kit (Cat. no. QG51106 Qiagen, Germany). Each sample was transferred to a 1.5 ml microcentrifuge tube and centrifuged (14,000 rpm, 2 min) to pellet the bacteria. Bacterial pellets were resuspended in 180 μl of the enzyme solution and incubated for at least 30 min at 37 °C. 20 μl of Proteinase K and 200 μl of Buffer AL were added, mixed by vortexing, and incubated. Each suspension was then incubated at 56 °C for 30 min following a further 15 min at 95 °C. After heat incubation, the suspensions were briefly centrifuged to spin down the suspension. DNA was then extracted following the manufacturer's protocol and eluted with 30 μl Buffer AE, and centrifuged (8,000 rpm, 1 min). After a final centrifugation step, DNA concentrations were measured using Nano-Photometer Pearl (Implen GmbH, Munich, Germany) and samples stored at −20 °C until further analysis could be performed.

### Library construction and 16S rRNA sequencing

For two-step PCR amplification, adapter sequences were added to the V4 hypervariable regions of the 16S rRNA gene, providing sufficient information to taxonomically classify the microbial communities based on methods used in the Human Microbiome Project. Heterogeneity spacers of 1–3 base pairs were added to the first PCR primers to improve balanced base composition throughout the sequencing run.

After each PCR step, the amplicons were purified using the AMPure XP PCR Purification Kit (Beckman Coulter Genomics, Danvers, MA, USA) and DNA quantified according to the manufacturers' instructions—using the Qubit dsDNA HS Assay Kit (Qubit, Thermo Fisher Scientific, Waltham, MA, USA) and Qubit 3.0 Fluorometer (Qubit, Thermo Fisher Scientific). Library Quantification Kit for Illumina (Kapa Biosciences, Woburn, MA, USA) was used for library quantification. Finally, the library was combined with 20% Illumina's internal PhiX control library (v3) (Illumina, San Diego, CA, USA), targeted for a cluster density of 800–950 K clusters/mm^2^, and sequenced in paired-end MiSeq mode for 250 runs. After sequencing, the MiSeq instrument performed image analysis, base calling, and data quality assessment; the detailed procedure was the same described in our previous study (Cheng et al., 2022).

Sequencing reads from different samples were identified and separated using a specific barcode at the 5' end of the sequence (two mismatches allowed). Sequencing data were processed using the Nextflow/Ampliseq version 2.7.0 pipeline (Straub et al., 2020), for read quality control, trimming and filtering (with parameters maxEE = c (2, 2), truncQ = 2, minLen = 50, maxLen = Inf), error rate learning and ASV inference, paired read assembly, chimera removal, and taxonomic assignment (using SILVA database version 138) (Yilmaz et al., 2014) (Figure 1B).

### Computational and statistical analysis

Data were analyzed using R software version 4.1.0 (The R Project for Statistical Computing, Vienna, Austria). Categorical variables are presented as frequency and percentage and were analyzed using the chi-squared test to determine significance. For non-parametric distribution data, differences between groups were assessed using the Wilcoxon-Mann-Whitney test. To explore the association between bacterial community and factors related to individuals, Python 3.10, bioinformatics, and scikit-learn version 0.1.4, alpha diversity analysis (Shannon index and Simpson's evenness index) was performed, and biodiversity between groups was compared.

Principal coordinate analysis (PCoA) was conducted using unweighted Bray-Curtis distances to assess differences in microbial community composition between groups based on amplicon sequence variants (ASVs). The Bray-Curtis distance matrix was calculated using the vegdist function in the vegan package. To visualize community composition, the first two PCoA axes (PC1 and PC2) were extracted and plotted using ggplot2.

Permutational multivariate analysis of variance (PERMANOVA) (Anderson, 2017), as implemented in the adonis2 function from the vegan package, was employed to evaluate the significance of the between-group differences. The PERMANOVA results were adjusted for multiple testing using the false discovery rate (FDR) method, ensuring robustness of the *p*-values (adjusted *p* < 0.05 was considered statistically significant). Additionally, analysis of similarities (ANOSIM) was performed to test if differences between groups were greater than within-group differences. Both analyses were based on 999 permutations.

To further assess the association between microbial composition and clinical variables, redundancy analysis (RDA) was performed using the rda function from the vegan package. The response matrix (microbial relative abundances) was transformed using the Hellinger method (decostand) to reduce the influence of rare taxa. Explanatory variables were encoded as numeric where necessary and filtered to remove columns with missing values. The RDA model (Capblancq and Forester, 2021) was fitted to the transformed response matrix with explanatory variables as predictors, and the results were visualized using ggplot2. The first two RDA axes (RDA1 and RDA2) were extracted, and sample groupings were highlighted using ellipses. Arrows representing explanatory variables were added to illustrate their contribution to the variation in microbial community structure.

To further explore differentially abundant taxa between groups, Linear Discriminant Analysis Effect Size (LEfSe) (Segata et al., 2011) was applied with an α significance level of 0.05 and a logarithmic LDA score threshold of 2.0. The phyloseq package was used to handle the abundance and metadata, while differentially abundant taxa were visualized using the ggplot2 and ggpubr packages. The significance of taxa in explaining differences between groups was visually represented using stacked bar plots and correlation matrices. Additionally, the relationship between PCoA axes (PC1, PC2) and clinical indicators such as mNUTRIC, APACHE II, and SOFA scores was assessed using the corrplot function (pearson correlation) to display correlations.

Logistic regression analysis was employed to identify microbial taxa features associated with group classification. Regression coefficients, confidence intervals, and *p*-values were computed, and significant features were visualized with error bars. To assess the prevalence of each microbial species, the detection rate was calculated as the percentage of samples in which the relative abundance exceeded a predefined threshold 0.1 (10%). Prevalence was compared between groups, with results visualized using heatmaps (Figure 1C).

### Development of explainable AI models

Data analysis and machine-learning model training were performed using Python 3.10 with the scikit-learn (v1.4.2) and bioinformatics libraries. Various machine-learning algorithms were trained to classify and predict corticosteroid response based on microbial community features.

To prevent overfitting and ensure methodological rigor, all feature selection and model optimization procedures were performed exclusively within the training set, maintaining a patient-level split (70% training, 30% testing) using the train_test_split function to avoid data leakage between sets. Random seeds were fixed for reproducibility.

The Random Forest (RF) classifier was optimized using a randomized search strategy (RandomizedSearchCV) to identify the best hyperparameters. The search space included the number of estimators (n_estimators: 30–100), maximum features (max_features: {‘log2', ‘sqrt'}), tree depth (max_depth: 1–10), minimum samples for node splitting and leaf formation (min_samples_split: 2–10; min_samples_leaf: 1–9), and bootstrap sampling (bootstrap: {True, False}). A total of 2,000 randomized search iterations were conducted with 5-fold cross-validation, optimizing for the area under the receiver operating characteristic curve (ROC-AUC). Class imbalance was handled using class_weight=“balanced”. The final model was evaluated on the independent test set, and ROC curves were plotted to visualize discrimination performance. To enhance interpretability, feature-importance rankings were calculated based on the mean decrease in Gini impurity for the selected microbial taxa and clinical indices. The formula of the Gini index is:


$$\begin{array}{l} Gini\left(D\right)=\overset{n}{\underset{i=1}{\sum }}p_{i}^{2} \end{array}$$

where D is the dataset, *n* is the number of classes in *D*, and *p*_*i*_ is the probability of class *i*. If a dataset *D* is split on feature *A* into subsets *D*_*i*_, the Gini index give the split on feature *A* is:


$$\begin{array}{l} Gini_{A}\left(D\right)=\underset{i}{\sum }\frac{\left|D_{i}\right|}{\left|D\;\right|}Gini\left(D_{i}\right) \end{array}$$

And the reduction of impurity is:


$$\begin{array}{l} \Delta Gini\left(A\right)=Gini\left(D\right)-Gini_{A}\left(D_{i}\right) \end{array}$$

The K-Nearest Neighbors (KNN) model was similarly trained using a randomized search to determine the optimal number of neighbors (n_neighbors), weighting function (weights), distance metric (metric), and Minkowski power parameter (p). The best KNN model was selected according to ROC-AUC performance using 5-fold cross-validation.

The Support Vector Machine (SVM) model was optimized via randomized search for hyperparameters including the regularization parameter (C), kernel type (kernel), gamma (gamma), polynomial degree (degree), and class weight (class_weight), with 5-fold cross-validation.

For all models, the roc_auc_score metric was used to evaluate test-set performance, and ROC curves were plotted to visually assess discriminatory ability. Additional classification metrics including precision, recall, and F1-score were calculated for comprehensive performance evaluation. All trained models were saved using joblib.dump() for reproducibility.

The final machine-learning models (RF, KNN, and SVM) were compared based on their ROC-AUC scores and the stability of performance across different random states, with the best-performing model selected for further clinical validation and deployment (Figure 1D).

## Results

### Demographic analysis and clinical characteristics of steroid treatment in SCAP patients

This study analyzed 200 SCAP patients, with 129 receiving corticosteroids (Steroid [+] group) and 71 not receiving corticosteroids (Steroid [–] group) (Table 1). The groups had comparable demographic characteristics, including age (73.2 ± 12.4 vs. 72.7 ± 12.7 years, *p* = 0.786) and gender distribution (*p* = 0.241), with a slightly higher proportion of males in both groups. Smoking prevalence was also similar (45% vs. 55%, *p* = 0.285).

**Table 1:** Demographic characteristics, severity scores, comorbidities, and clinical outcomes of pneumonia patients in the ICU.

**Variable**	**Steroid (+)**	**Steroid (-)**	***P*-value**
Number of Patients	129	71	-
**Age (years)**	73.2 ± 12.4	72.7 ± 12.7	0.786
Gender			
Male	86 (67%)	53 (75%)	-
Female	43 (33%)	18 (25%)	0.241
Smoking			
Yes	59 (46%)	39 (56%)	-
No	70 (54%)	31 (44%)	0.285
Mortality			
Survival	91 (71%)	54 (76%)	-
Deceased	38 (29%)	17 (24%)	0.403
COPD			
Yes	40 (31%)	14 (20%)	-
No	89 (69%)	57 (80%)	0.085
SOFA score			
Day 1	8.4 ± 3.1	8.6 ± 3.2	0.698
Day 3	7.2 ± 3.4	7.0 ± 3.4	0.738
Day 7	6.3 ± 3.5	5.9 ± 2.9	0.469
**mNUTRIC score**	6.1 ± 1.5	6.0 ± 1.5	0.538
**APACHE II score**	28.0 ± 5.9	25.7 ± 6.1	0.009^*^

The table summarizes key characteristics of 200 pneumonia patients, stratified by systemic corticosteroid treatment status. Patients received steroids were classified as Steroid (+) group, and the others were classified as Steroid (–) group. Data include demographics, severity scores, comorbidities, and clinical outcomes. Continuous variables were analyzed using *t*-tests, and categorical variables were analyzed using chi-square tests. Statistically significant results are marked (^*^p < 0.05).

Although mortality rates (29.5% vs. 23.9%, *p* = 0.403) and COPD prevalence (31.0% vs. 19.7%, *p* = 0.085) were slightly higher in the Steroid [+] group, these differences were not statistically significant. Severity scores, including the SOFA and mNUTRIC scores, showed no significant differences between groups. However, the APACHE II score was significantly higher in the Steroid [+] group (28.1 ± 5.88 vs. 25.8 ± 6.05, *p* = 0.010), indicating greater disease severity.

### Comparative analysis of pulmonary pathogen detection: next-generation sequencing vs. traditional culture methods

This analysis compares microbial detection at three time points (D1, D3, and D7) using traditional bacterial culture (TBC) and next-generation sequencing (NGS) to evaluate the strengths and limitations of each method in identifying respiratory pathogens. Traditional culture identified only a limited number of pulmonary pathogens, whereas NGS significantly increased detection rates.

**Figure 2:**
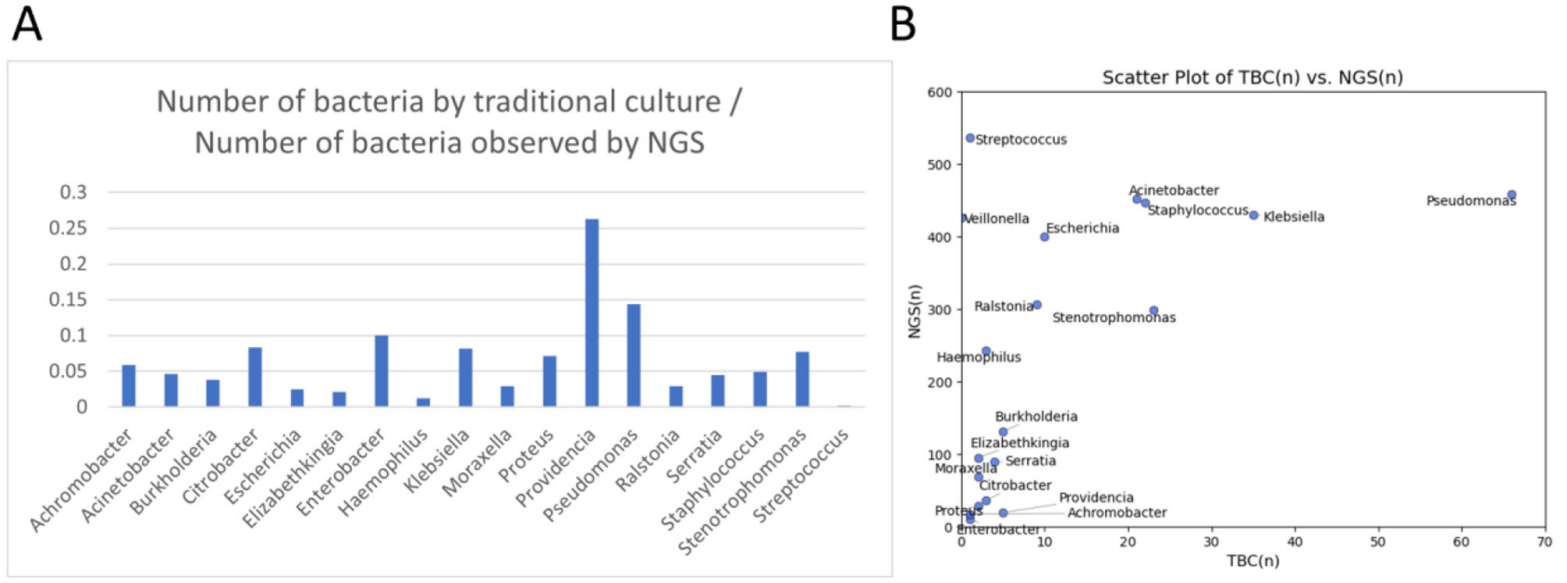
Comparison of pathogen identification by NGS and traditional bacterial culture (TBC) method. **(A)** The TBC/NGS ratio. The value closer to 1 indicates that both methods detected the pathogen at similar frequencies. Conversely, the value of 0 indicates that the pathogen was detected by NGS but not by traditional culture methods. **(B)** Scatter plot comparing the number of pathogens identified by NGS and traditional culture method.

Figure 2 illustrates the detection ratio between the two methods: a ratio close to 1 indicates similar detection frequencies, whereas a ratio of 0 means the pathogen was identified only by NGS. The table quantifies detection rates, assuming all 200 patients survived until Day 7. If a particular bacterium was detected in every patient at each time point, the maximum value would be 600 (200 × 3). The TBC/NGS ratio reflects detection consistency between methods, with higher values indicating agreement and lower values highlighting NGS's superior sensitivity. These findings underscore the advantages of NGS in identifying respiratory pathogens that may be missed by traditional culture methods (Supplementary Tables 1–4).

### Characterization of pulmonary microbiota in patients with and without steroid treatment

#### Microbial differences on day 1 between steroid-treated and untreated patients

**Figure 3:**
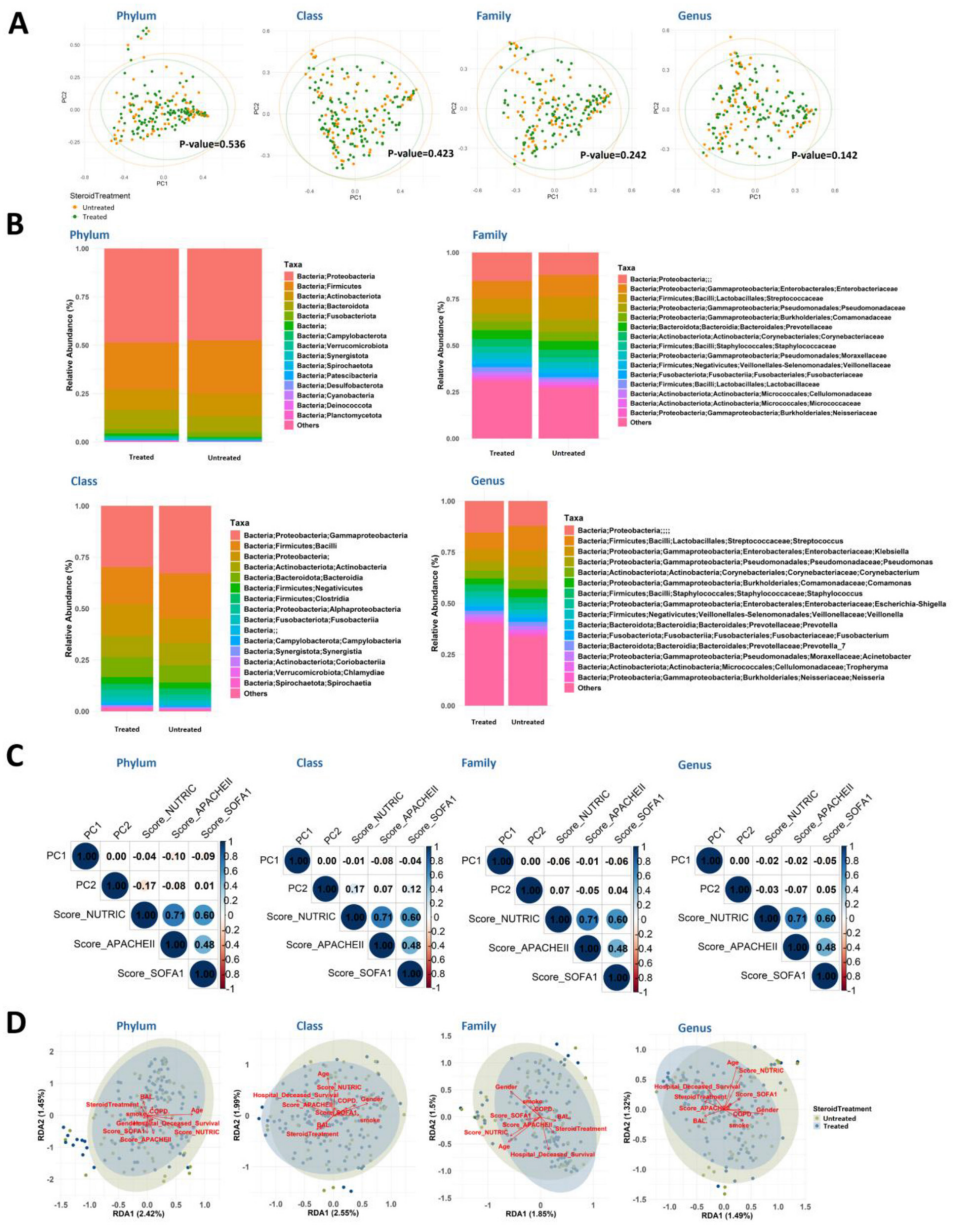
Global microbiome composition and its associations with clinical indicators for corticosteroid treatment. **(A)** PCoA plots illustrate the microbiome composition differences between Steroid (+) and Steroid (–) groups across various taxonomic levels. Adonis test p-values indicate statistical significance. **(B)** Average stacked bar charts displaying the relative abundance of the top 15 taxa at each taxonomic level for the two groups, with less abundant taxa grouped as “Others.” **(C)** Pearson correlation coefficients between PC1/PC2 from PCoA at different taxonomic levels and clinical indicators. **(D)** RDA plot at the phylum level highlight the relationship between clinical indicators (e.g., mNUTRIC, APACHE II, SOFA scores) and microbiome composition. Angles between vectors indicate the strength and direction of correlations.

Next-generation sequencing (NGS) revealed differences in pulmonary microbiota composition between corticosteroid-treated and untreated SCAP patients. Principal Coordinate Analysis (PCoA) on Day 1 (D1) showed no statistically significant global differences between the two groups at the phylum, class, family, or genus levels (Phylum: *p* = 0.536; Class: *p* = 0.423; Family: *p* = 0.242; Genus: *p* = 0.142; Figure 3A).

Taxonomic comparisons showed lower relative abundances of key bacterial groups in the Steroid [+] group, including Firmicutes, Gammaproteobacteria, Bacilli, Enterobacteriaceae, Streptococcaceae, Pseudomonadaceae, *Streptococcus, Klebsiella*, and *Pseudomonas* (Figure 3B). Although these differences were not statistically significant, they suggest a potential impact of corticosteroid treatment on microbiota composition. Additionally, alpha diversity (Shannon index, Simpson's evenness index, and observed OTUs) showed a slight downward trend over time in both groups, though without statistical significance (Supplementary Figure 1).

#### Associations between microbiota and clinical indicators

To explore correlations between microbiota composition and clinical indicators, redundancy analysis (RDA) and principal component analysis (PCA) were performed. Across all taxonomic levels, PC1 and PC2 showed weak correlations with clinical indicators. At the phylum level, PC2 was negatively correlated with the mNUTRIC score (-0.17), while at the class level, PC2 showed a weak positive correlation (0.17). Similar weak associations were observed at the family and genus levels, with correlation coefficients ranging from −0.06 to 0.07 (Figure 3C).

Furthermore, RDA analysis showed that clinical severity scores (mNUTRIC, APACHE II, and SOFA) formed large angles with the corticosteroid treatment vector, suggesting limited direct correlation between overall microbiota shifts and corticosteroid use (Figure 3D). However, specific taxa demonstrated stronger associations with clinical outcomes, as discussed below.

### Distinct microbial composition in survivors and non-survivors of corticosteroid-treated SCAP patients

#### Microbiome differences between survivors and non-survivors

Principal Coordinate Analysis (PCoA) on Day 1 (D1) showed no significant differences in pulmonary microbiota composition between corticosteroid-treated and untreated SCAP patients. However, within the corticosteroid-treated cohort, survivors and non-survivors exhibited distinct microbial shifts by Day 7 (D7). The non-survivor (NS) group had significantly higher SOFA, mNUTRIC, and APACHE II scores, indicating worse clinical outcomes (Table 2).

**Table 2:** Clinical characteristics and outcomes of “survivor” group (S) vs. “non- survivor” group (NS) in corticosteroid treatment.

**Variable**	**Survivor**	**Non-Survivor**	***p*-value**
Number of Patients	91	38	-
Gender			
Male	62 (68%)	24 (63%)	-
Female	29 (32%)	14 (37%)	0.249
SOFA score			
Day 1	8.0 ± 3.1	9.2 ± 3.0	0.442
Day 3	6.9 ± 3.2	7.8 ± 3.7	0.180
Day 7	5.5 ± 3.0	7.2 ± 4.0	0.019^*^
**mNUTRIC score**	5.9 ± 1.5	6.6 ± 1.3	0.016^*^
**APACHE II score**	27.4 ± 5.9	29.7 ± 5.6	0.040^*^

Comparison of clinical scores, comorbidities, and outcomes between the two groups. Continuous variables were analyzed using *t*-tests, and categorical variables were analyzed using chi-square tests. Statistically significant results are marked (^*^p < 0.05).

**Figure 4:**
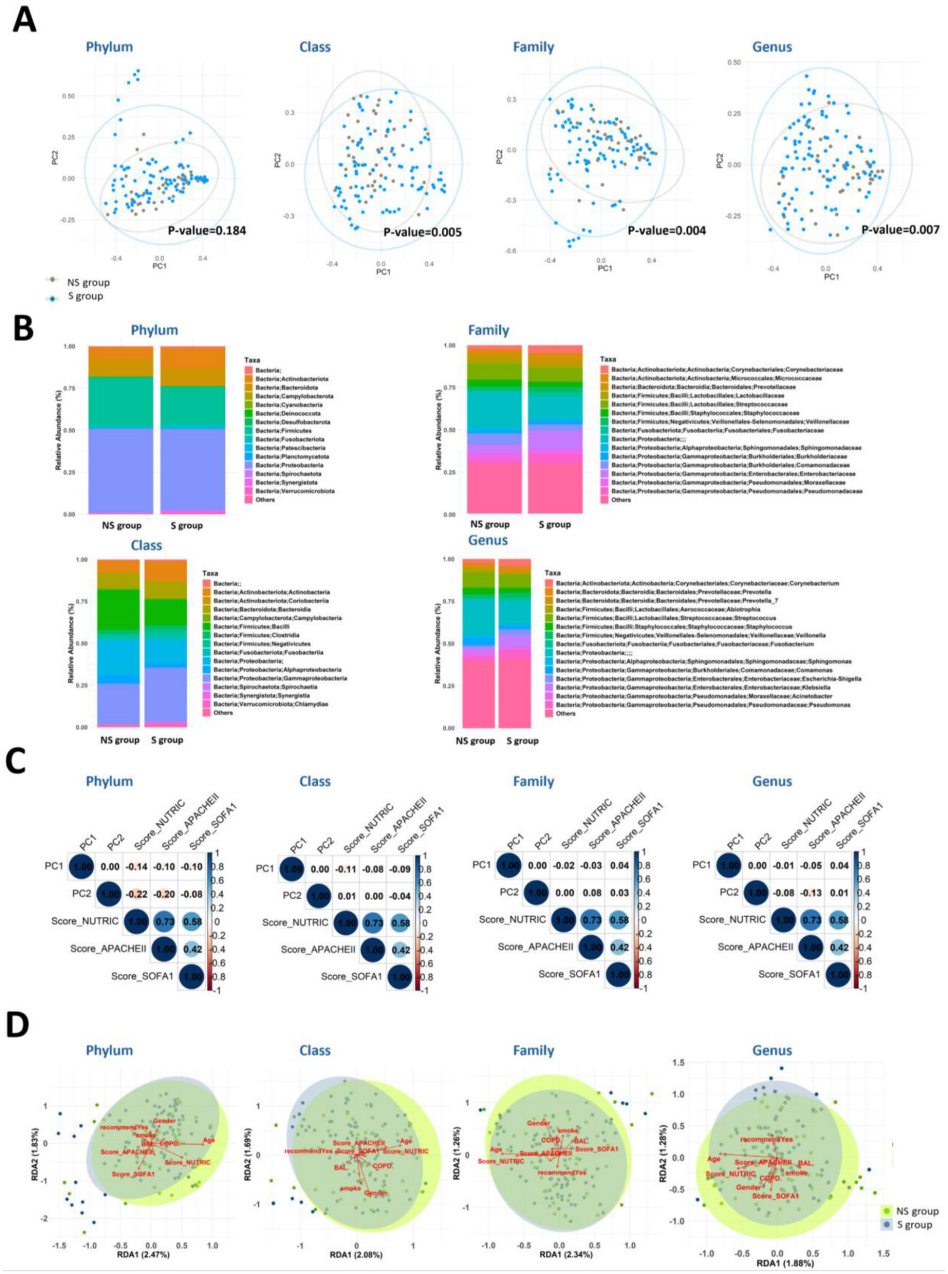
Microbiome composition differences between Survivor (S) and Non-Survivor (NS) groups. **(A)** PCoA plots show the global microbiome composition differences at various taxonomic levels. Significant differences were observed at the class (*p* = 0.005), family (*p* = 0.004), and order (*p* = 0.007) levels using the Adonis test, while differences at the phylum level were not statistically significant (*p* = 0.184). **(B)** Average stacked bar charts depict the relative abundance of the top 15 taxa at each taxonomic level, with less abundant taxa grouped as “Others.” **(C)** Pearson correlation coefficients between PC1/PC2 from PCoA and clinical indicators are presented for various taxonomic levels. **(D)** RDA plots illustrate relationships between clinical indicators (e.g., mNUTRIC, APACHE II, SOFA scores) and microbiome composition at the phylum level, where larger angles with the corticosteroid treatment vector suggest negative correlations.

PCoA revealed significant differences in microbiome composition at the class, family, and genus levels (Phylum: *p* = 0.184; Class: *p* = 0.005; Family: *p* = 0.004; Genus: *p* = 0.007; Figure 4A). Survivors (S group) had higher relative abundances of Actinobacteria and Gammaproteobacteria, while non-survivors (NS group) showed increased Campylobacteria and Alphaproteobacteria (Figure 4B). Alpha diversity analysis indicated a slight downward trend in Shannon index and observed OTUs over time in both groups, though these changes were not statistically significant (Supplementary Figure 2). Simpson's evenness index remained stable throughout the study.

#### Associations between microbiota and clinical indicators

At the phylum level, PC1 and PC2 exhibited weak negative correlations with the APACHE II and mNUTRIC scores (Figure 4C). RDA analysis showed that APACHE II and SOFA scores formed smaller angles with survival outcomes, suggesting potential interactions (Figure 4D). At the class, family, and genus levels, correlations were weaker, with vectors nearly orthogonal, indicating minimal relationships between clinical indicators and survival (Figure 4C). Despite weak direct correlations, microbiome composition consistently differed between survivors and non-survivors across all levels in PCoA analyses (Figure 4A), suggesting that microbial patterns may offer additional insights beyond traditional clinical indicators.

#### Key microbial taxa associated with corticosteroid response

**Figure 5:**
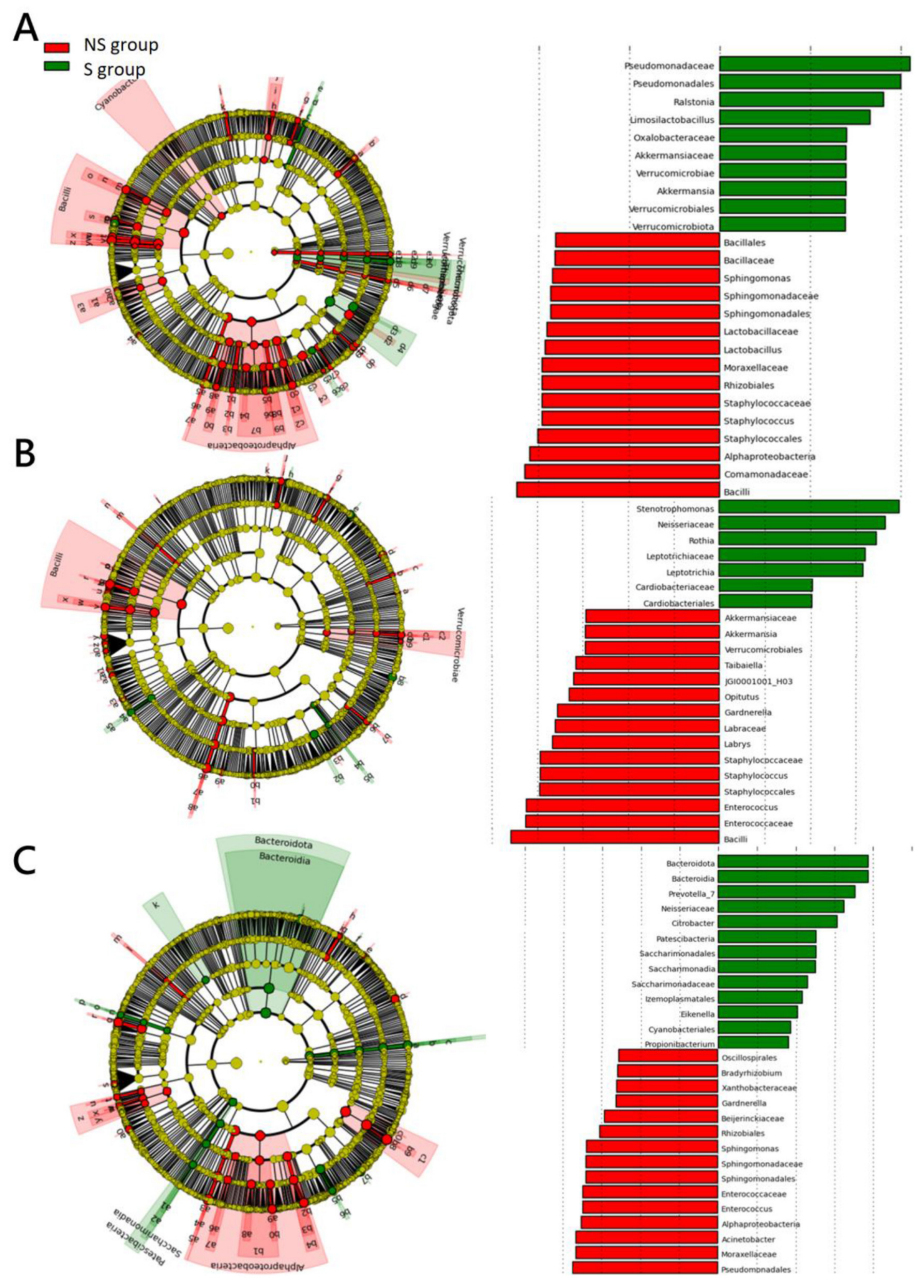
LEfSe analysis of microbial trends in survivors (S) and non-survivors (NS) receiving corticosteroid. **(A–C)** represent analysis results from Day 1, Day 3, and Day 7, respectively. Bar charts display the top 15 differentially abundant species, with green bars indicating higher abundance in survivors who responded to corticosteroid treatment and red bars indicating higher abundance in non-survivors who did not.

LEfSe analysis identified distinct microbial taxa linked to survival outcomes. Non-survivors exhibited higher abundances of Bacilli, Ruminococcaceae*, Sphingomonas, Vibrio, Oscillibacter*, and *Enterococcus* (Figure 5), suggesting their potential associations of poor corticosteroid response.

In the non-survivor (NS) group, several species persisted from Day 1 to Day 7. Bacilli, Vibrionaceae*, Vibrio*, and *HT002* were present on both D1 and D3, while *Oscillibacter*, Thermales, Thermaceae, Coriobacteriaceae, Enterococcaceae, and *Enterococcus* were more prominent on D3 and D7. Species such as Alphaproteobacteria, Moraxellaceae, Ruminococcaceae, Staphylococcales, Staphylococcaceae, Caulobacterales, and *Sphingomonas* were consistently detected on D1 and D7. These findings imply that variations in Alphaproteobacteria and Bacilli may serve as potential microbial indicators of corticosteroid responsiveness.

#### Trends in differentially abundant taxa and treatment response

**Figure 6:**
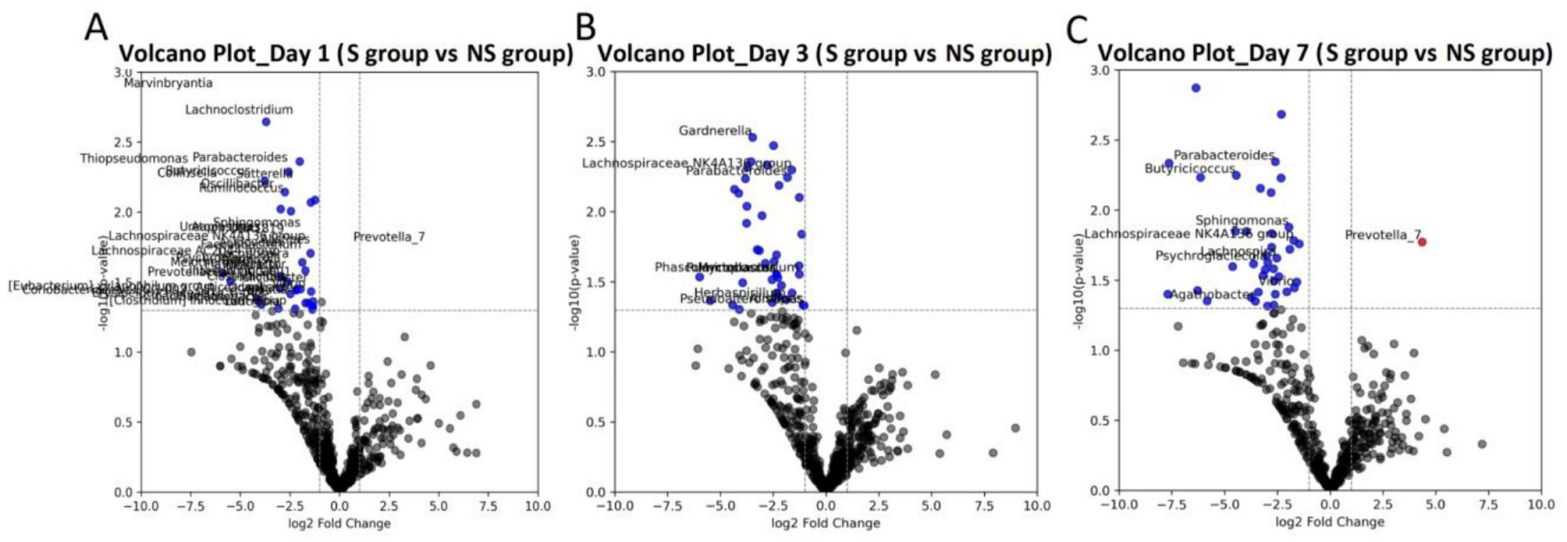
Volcano plots illustrating microbial differences between survivor (S) and non-survivor (NS) groups. **(A–C)** represent the analysis results from Day 1, Day 3, and Day 7, respectively. Red dots represent species with log_2_(FC) > 1 and p-value < 0.05, while blue dots represent species with log_2_(FC) < −1 and *p*-value < 0.05.

Microbial composition trends correlated with survival outcomes in SCAP patients. In the survivor group, multiple taxa, including *Parabacteroides, Ruminococcus, Thiopseudomonas*, and *Vibrio*, were consistently downregulated across time points (Figure 6), which may reflect their association with a more favorable outcome.

**Figure 7:**
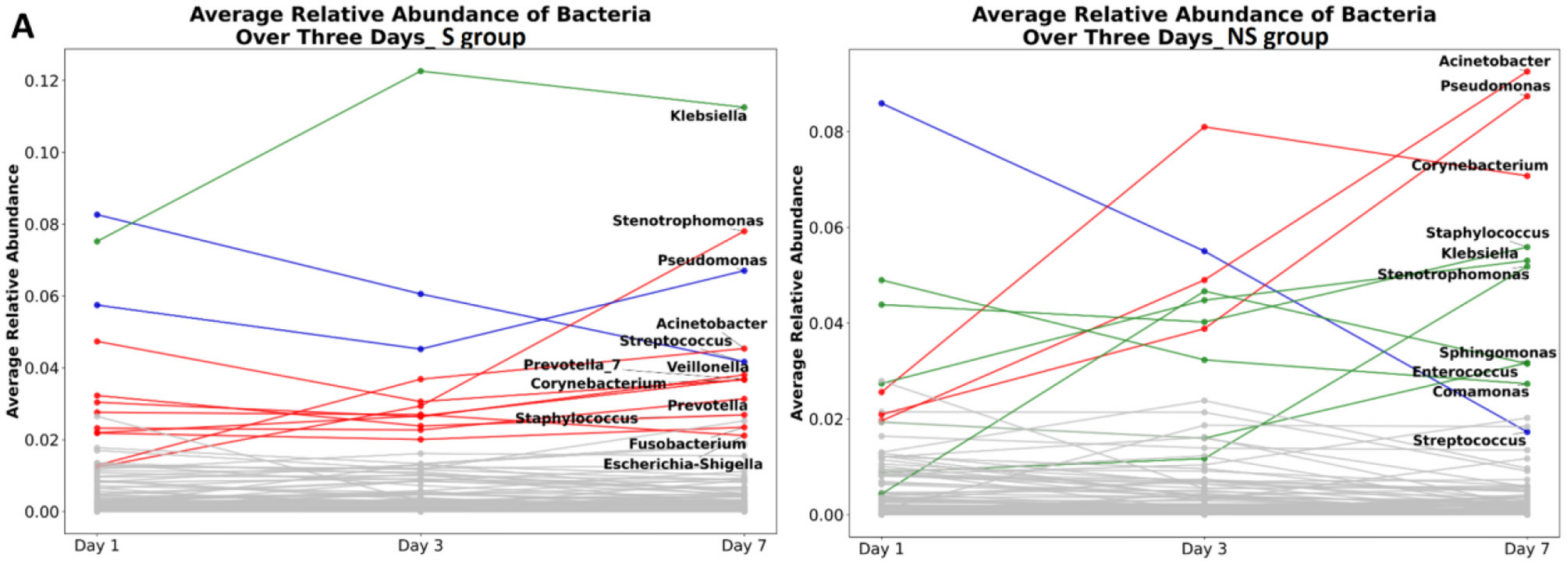
Temporal trends in relative abundance of microbial genera over seven days between Survivor (S) and non-survivor (NS) group. **(A, B)** represent the results from Day 1, Day 3, and Day 7 for survivor and non-survivor groups, respectively.

Longitudinal microbial trends further distinguished survivors from non-survivors (Figure 7). In the NS group, *Acinetobacter* and *Pseudomonas* exhibited continuous increases from D1 to D7, while *Streptococcus* showed a marked decline. Conversely, *Klebsiella* remained consistently abundant in survivors. These findings suggest that microbial dynamics may play a meaningful role in influencing treatment outcomes and patient recovery, underscoring the potential value of microbiome-informed strategies in precision medicine.

### Explainable AI models for predicting corticosteroid response in SCAP patients

**Figure 8:**
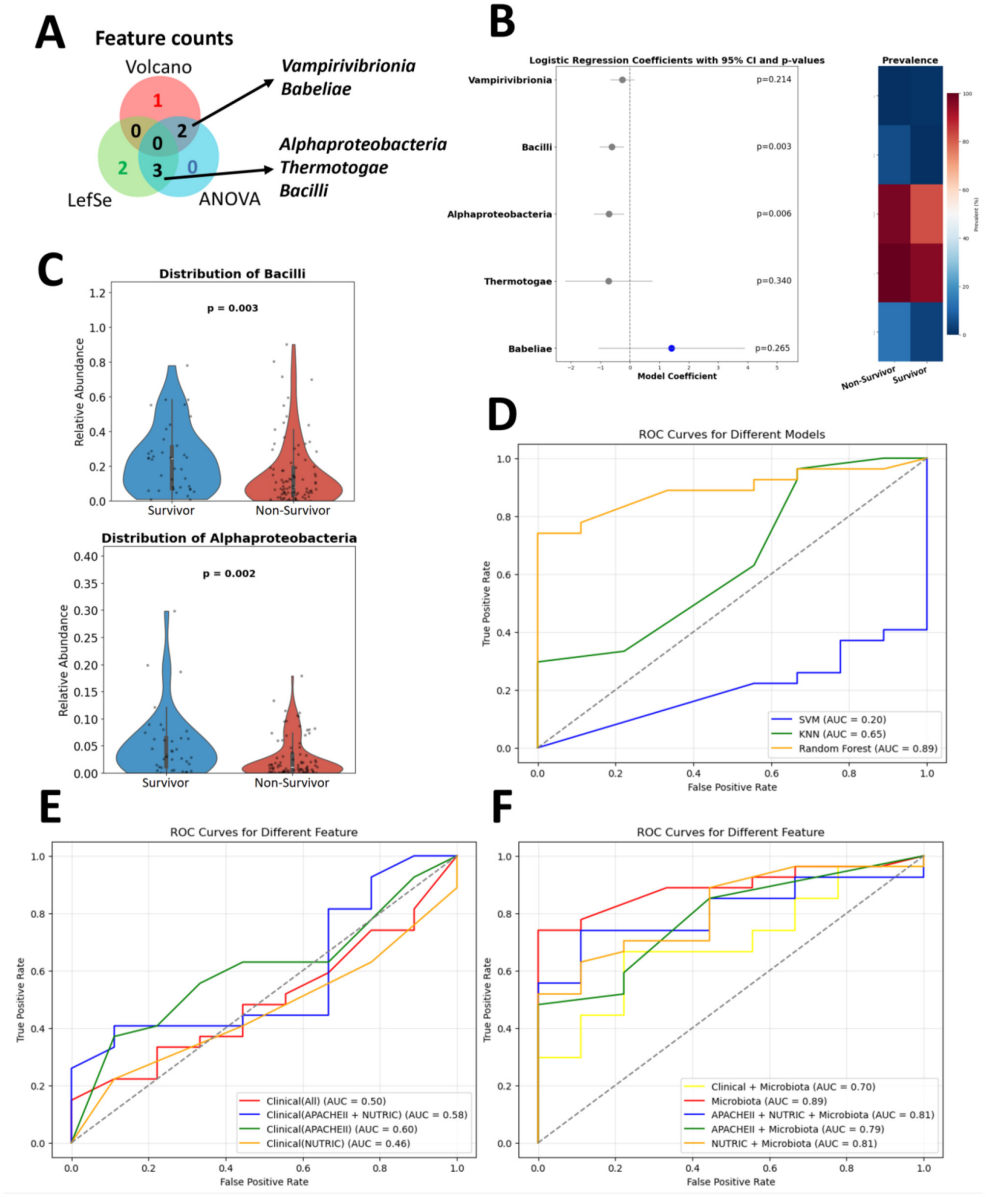
Identification and validation of microbial class-level features predictive of corticosteroid treatment response. **(A)** Venn diagram showing microbial classes identified by at least two out of three feature selection methods (LEfSe, ANOVA, and differential abundance analysis), highlighting shared features. **(B)** Logistic regression analysis results, identifying five candidate microbial classes, with Bacilli and Alphaproteobacteria showing statistically significant differences (*p* < 0.05) between groups. **(C)** Violin plots comparing the abundances of Bacilli and Alphaproteobacteria between the survivor and non-survivor groups. Both classes are significantly enriched in the non-survivor group. **(D)** ROC curves for three machine learning models trained using Bacilli and Alphaproteobacteria as predictive features. The random forest (RF) model achieves the best performance with an (AUC = 0.89), followed by KNN (AUC = 0.65) and SVM (AUC = 0.20), demonstrating the utility of these features for treatment response prediction. **(E)** Clinical indicators were used to predict corticosteroid treatment responses using RF model. **(F)** Combinations of 2 key microbiota and clinical indicators were used to predict corticosteroid treatment responses using RF model. While conventional clinical indicators, including APACHE II and mNUTRIC scores, provide limited predictive value, microbiota-based features significantly enhance model performance, suggesting that microbial composition may serve as a more sensitive biomarker for treatment response.

To predict corticosteroid response in SCAP patients, multiple feature selection approaches, including LEfSe analysis, ANOVA, and differential abundance analysis, were used to identify key microbial taxa. Taxa selected by at least two of the three methods were considered for predictive modeling (Figure 8A). Logistic regression highlighted five candidate taxa, with Bacilli and Alphaproteobacteria showing statistically significant differences between survivors and non-survivors (Figure 8B). Violin plots confirmed that both taxa were significantly more abundant in non-survivors (Figure 8C).

Machine learning models, including random forest (RF), k-nearest neighbors (KNN), and support vector machine (SVM), were trained using Bacilli and Alphaproteobacteria as predictive features. Among these, the RF model demonstrated the highest predictive performance (AUC = 0.89), significantly outperforming KNN (AUC = 0.65) and SVM (AUC = 0.20) (Figure 8D).

The RF model was further evaluated using different feature combinations of microbiota and clinical indicators. When only clinical variables were used, the model performed poorly (AUC = 0.50), with slight improvement for significant markers (APACHE II: AUC = 0.60; mNUTRIC: AUC = 0.46; combined: AUC = 0.58) (Figure 8E). In contrast, using only the two key microbial taxa yielded the highest predictive accuracy (AUC = 0.89). Adding clinical features slightly reduced performance (AUC = 0.81 when combining APACHE II and mNUTRIC scores; AUC = 0.70 when including all clinical indicators).

When integrating both microbial and clinical variables, Bacilli (0.324) and Alphaproteobacteria (0.299) together accounted for approximately two-thirds (65%) of the total feature importance, whereas clinical scores contributed less (APACHE II: 0.23; mNUTRIC: 0.15). This indicates that microbial features remained the dominant predictors of corticosteroid response, even when combined with established clinical indices (Figure 8F). Collectively, these findings highlight the strong predictive power and interpretability of microbiota-based machine learning models, underscoring their potential in precision medicine for optimizing corticosteroid treatment in SCAP patients.

## Discussions

Advances in high-throughput sequencing, especially metagenomic next-generation sequencing (mNGS), have greatly enhanced our ability to profile the lung microbiome and identify pathogens. While specific microbial profiles have been shown to predict prognosis in patients with severe pneumonia, the effects of SG therapy on the lung microbiome in SCAP remain unclear. Indeed, there is very little known about how SG affects lung microbial communities even in other respiratory diseases. By contrast, the many investigations of ICS in conditions such as asthma, COPD and rhinitis (Hartmann et al., 2021; Toraldo and Conte, 2019; Toraldo et al., 2022) show microbial-community shifts, but such results cannot be validly extrapolated to SG therapy in SCAP given the markedly different pharmacology, systemic scope and disease severity. Furthermore, current research indicates that the efficacy of SG in SCAP patients remains inconsistent (Meduri et al., 2022; Iqbal et al., 2020; Dequin et al., 2023; Wu et al., 2023; Nora et al., 2020; Ricard and Messika, 2015; Saleem et al., 2023; Torres et al., 2015; File, 2015): many studies focus on survival endpoints but do not examine whether or how changes in the lung microbiome mediate or modify that response. Some studies report that SCAP patients exhibit more microbiome dysbiosis of the respiratory microbiota than non-SCAP cases, often characterized by markedly reduced microbial diversity and shifts toward pathogen-dominated taxa (Vidaur et al., 2025; Yang et al., 2024), yet these investigations do not link SG therapy to microbial shifts. Consequently, our study aims to fill this important research gap by combining longitudinal profiling of the sputum microbiome with explainable machine-learning methods to identify microbial signatures that may predict which patients with SCAP are most likely to benefit from SG therapy, thereby advancing the potential for precision-guided corticosteroid use.

Our results showed that while overall microbial composition, assessed through PCoA, did not significantly differ between corticosteroid-treated and untreated groups, notable differences emerged when survivors and non-survivors were compared. In particular, higher abundances of Alphaproteobacteria and Bacilli were observed in non-survivors, hinting at a possible association with corticosteroid treatment response. These findings underscore the potential relevance of microbial composition for patient prognosis and suggest that assessment of the microbiota may merit consideration when evaluating corticosteroid therapy.

Temporal trends further illustrated the dynamic nature of microbial responses to corticosteroids. In the treatment group, bacterial abundance initially increased but declined by Day 7, whereas the untreated group exhibited a sustained increase. Although confirmed via LEfSe and differential-abundance analyses, these patterns should be interpreted cautiously as they may reflect differential treatment outcomes rather than causal microbial shifts. Notably, among corticosteroid-treated patients, deceased individuals had elevated Proteobacteria, particularly Alphaproteobacteria, consistent with previous research linking Proteobacteria to inflammatory diseases such as asthma and inflammatory bowel disease. This further underscores its potential role in poor prognosis and corticosteroid responsiveness. While corticosteroids are known to affect the microbiome in respiratory diseases, their specific impact on SCAP microbiota remains largely unexplored. Future research should elucidate the relationship between specific microbial communities and corticosteroid recommendations in SCAP.

The RF-based model, incorporating Alphaproteobacteria and Bacilli as biomarkers, achieved the highest predictive accuracy, demonstrating the utility of microbiota-based models in clinical decision-making. The potential mechanistic relevance of Bacilli and Alphaproteobacteria in modulating corticosteroid responsiveness merits consideration. Members of the Bacilli class (for example, genera such as *Streptococcus* and *Enterococcus*) may influence the overall functionality of the microbial community through the production of extracellular polysaccharides and the promotion of taxa producing short chain fatty acids (SCFAs), leading to altered SCFA levels or signaling, which in turn may modulate the host's immune and epigenetic landscape, thereby potentially altering sensitivity to systemic corticosteroids, given that steroid responsiveness depends on the host's baseline immune-inflammatory state and downstream signaling networks (Ramos et al., 2021; Tarique et al., 2024; Li et al., 2024b). Conversely, members of the Alphaproteobacteria class, including genera like *Sphingomonas* and *Rhodobacter*, harbor stress-response systems and cellular metabolic adaptations (such as oxidative stress resistance and membrane lipid modifications) that might attenuate the host's steroid-mediated suppression of inflammation or influence local steroid metabolism (Li et al., 2024b; Licht et al., 2020; Kaczmarczyk et al., 2011; Francez-Charlot et al., 2015). Collectively, these observations argue that variations in the abundance or activity of these bacterial classes may influence the host's functional response to systemic corticosteroids, and hence they represent plausible biological links underlying the differential steroid responsiveness observed in our cohort. Additionally, the association between Proteobacteria and mortality aligns with previous findings linking it to inflammation and poor outcomes (Dicker et al., 2021), reinforcing the microbiome's role in corticosteroid responsiveness. These findings highlight the potential of microbiota composition as a predictive marker for corticosteroid treatment in SCAP.

While conventional clinical indicators such as APACHE II and mNUTRIC scores provided limited predictive value, microbiota-based features significantly enhanced model performance. The decline in AUC when combining microbiota with multiple clinical indicators suggests potential redundancy or noise introduced by additional variables. Despite integrating widely used clinical severity indices (APACHE II and mNUTRIC), microbial taxa still contributed the majority of model importance (≈65%). This finding suggests that host-independent microbial signatures contain predictive information beyond conventional clinical scores, reinforcing the value of microbiome profiling in critical care. These results underscore the importance of integrating microbiome analysis into precision medicine strategies, enabling more accurate identification of corticosteroid responders and optimizing SCAP treatment. Further studies investigating host-microbe interactions and microbial metabolic pathways could clarify mechanisms contributing to treatment variability.

## Limitations and Future Directions

Despite its contributions, this study has several limitations. First, it was conducted in a single ICU, potentially limiting generalizability. Expanding the cohort to multiple centers with diverse patient populations would enhance result robustness. Second, while mNGS provided high-resolution microbial profiling, functional analysis of microbiota was not performed. Third, external validation of the machine learning model is required to assess its robustness across different cohorts. Establishing causal relationships between microbial shifts and corticosteroid responsiveness will require further experimental validation through *in vitro* and animal studies.

Future research should investigate how systemic glucocorticoids modulate host-microbiota interactions, including downstream immune-mediated responses. Clinical trials exploring personalized corticosteroid regimens guided by microbial biomarkers are warranted to establish the translational potential of these findings. Furthermore, longitudinal investigations across distinct disease stages will be essential in identifying critical temporal biomarkers and clarifying their relevance for treatment outcomes. From a practical standpoint, our predictive model could be implemented in an ICU decision-support environment via rapid mNGS (within 24–48 h of admission) to guide corticosteroid use or antibiotic stewardship. Such an approach aligns with emerging microbiome-based precision medicine paradigms in critical care. To ensure that our findings are broadly applicable, we are initiating a multicenter validation study that will incorporate host transcriptomic and immune-profiling data across diverse ICU environments. External multicenter validation is indispensable to confirm that the predictive microbiome signatures and corticosteroid-response model retain robustness and generalizability beyond our single-center cohort. Together, these steps will enhance not only mechanistic understanding but also the practical translation of microbiome-guided corticosteroid use in severe critical-care settings.

Furthermore, our study demonstrates that identifying specific sputum microbiome signatures to stratify adult patients with SCAP who are receiving SG carries substantial implications for low- and middle-income countries (LMICs). In settings where critical-care resources are exceedingly limited and the burden of pneumonia is disproportionately high, this approach offers clinicians a means to conserve scarce resources and avoid indiscriminate use of systemic steroids. By focusing administration on patients most likely to benefit, the strategy supports precision-medicine initiatives, mitigates unnecessary risk, and enhances cost-effectiveness within LMIC healthcare systems.

## Conclusion

This study provides preliminary evidence that sputum microbiome profiling may offer prognostic insight for SCAP patients undergoing systemic glucocorticoid therapy. Although overall microbiota composition did not differ significantly between SG-treated and untreated groups, we observed higher abundances of Bacilli and Alphaproteobacteria in non-survivors, and our proposed explainable AI prediction model suggested these taxa could serve as candidate biomarkers of treatment response. Temporal microbial trends further emphasize the need for adaptive treatment approaches. However, before this approach can underpin corticosteroid stewardship, independent validation in external cohorts and integration with host-response biomarkers are essential to provide a foundation for precision-medicine strategies in SCAP management, guiding future research on microbial influences in treatment efficacy.

## Data Availability

The datasets presented in this study can be found in online repositories. The names of the repository/repositories and accession number(s) can be found at: https://www.ncbi.nlm.nih.gov/, BioProject/PRJNA1213238.
