# Supplementary material for: Lung microbiome signatures and explainable predictive modeling of glucocorticoid response in severe community acquired pneumonia
Source: Front Microbiol. 2025 Nov 28;16:1706432. doi: 10.3389/fmicb.2025.1706432 (PMC12698617; doi:10.3389/fmicb.2025.1706432)
Supplement: Supplementary file 1 [file Data_Sheet_1.docx]

Impact of corticosteroid treatment on lung microbiome and in-hospital mortality in patients with severe community-acquired pneumonia

Yeong-Nan Cheng, Guan-Ting Chen, Wei-Chih Huang, Yen-Peng Chiu, Yun Tang, Hsien-Da Huang, Pin-Kuei Fu, Tzong-Yi Lee^*^

**ONLINE DATA SUPPLEMENT**

**Supplementary Results**

**Comparative Analysis of Pulmonary Pathogen Detection between Next-generation Sequencing and Traditional Culture Methods**

The data of 200 patients from D1 reveals a significant disparity between the detection capabilities of Culture and NGS (**Supplementary Table 2, Supplementary Figure 1**). NGS overwhelmingly identified a larger number of microbial species compared to Culture. NGS identified a considerably higher number of bacterial species compared to culture, with certain species, such as Streptococcus (181 cases), Veillonella (137 cases), and Escherichia (121 cases), predominantly detected by NGS alone. Few bacteria, such as Enterobacter (3 cases) and Citrobacter (1 case), were identified exclusively through culture. The overlap between methods was minimal, with Pseudomonas (23 cases), Klebsiella (16 cases), and Staphylococcus (9 cases) being among the few species detected by both approaches. This highlights the superior sensitivity and coverage of NGS in detecting these respiratory microbes.

The results of bacterial detection from D3 further reinforce the trend observed in the overall dataset, where NGS demonstrated superior sensitivity compared to traditional culture methods. NGS exclusively identified a vast majority of cases for several bacterial species (**Supplement Table 3**), including *Streptococcus* (166 cases), *Veillonella* (122 cases), and *Staphylococcus* (114 cases). While *Pseudomonas* and *Klebsiella* were among the few species detected by both methods (17 and 7 cases, respectively), culture alone detected only a minimal number of cases, such as *Achromobacter* (1 case) and *Enterobacter* (1 case). Notably, *Haemophilus* and Escherichia remained predominantly detectable via NGS, with almost no cases identified through culture. On Day 7, NGS continued to exhibit a markedly higher detection rate compared to traditional culture methods. The vast majority of bacterial species, including *Streptococcus* (140 cases), *Acinetobacter* (111 cases), and *Veillonella* (94 cases), were predominantly identified through NGS alone (**Supplement Table 4**). *Pseudomonas* and *Klebsiella* remained among the few species detected by both methods (24 and 12 cases, respectively), while culture alone detected only *Enterobacter* (2 cases) and *Ralstonia* (1 case). Notably, Escherichia and *Haemophilus*, which were previously detected at higher frequencies via NGS, showed a decline in cases by Day 7. Across all three time points, the consistent trend highlights the superior sensitivity of NGS over culture in identifying pulmonary microbiota. The progressive shifts in microbial composition suggest dynamic microbial changes over time, which may have implications for disease progression and treatment response in SCAP patients.

**Characterization of Pulmonary Microbiota in Patients With or Without Steroid Treatment**

***Identification of Key Microbial Taxa Between Patients Receiving and not Receiving Corticosteroid Treatment Using LEfSe Analysis***

LEfSe analysis revealed distinct microbial taxa between corticosteroid-treated and untreated groups across different days (**Supplementary Figure 3**). On both Day 1 and Day 3, the Steroid (-) group showed a higher abundance of taxa such as *Clostridium_innocuum group,* and *Clostridium sensu stricto 1* (at species level); *Veillonella, Aquabacterium, Enhydrobacter, Micrococcus, Actinobacillus, Psychrobacter, Belnapia, Olephilus,* and *Bacteroides* (at genus level); Veillonellaceae, Cellulomonadaceae, Bacteroidaceae, Micrococcaceae, Leptotrichiaceae, Propionibacteriaceae, Acidaminococcaceae, Rikenellaceae, Chroococcidiopsaceae, Olephilaceae, Nocardioidaceae, and Arcobacteraceae (at family level); Veillonellales_Selenomonadales, Cyanobacteriales, Acidaminococcales, Clostridiales, and Planctomycetes, (at order level); Negativicutes (at class level); Cyanobacteria (at phylum level)*.* Additional taxa, including Desulfobacterota, Desulfovibrionia, Desulfovibrionales, Desulfovibrionaceae, Marinifiaceae, and *Odoribacter* were consistently observed in the Steroid (-) group on these days. By the Day 7, the untreated group displayed increased diversity, with prominent taxa such as *Elusimicrobiota, Verrucomicrobiota,* and *Rhizorhapis*. In contrast, the Steroid (+) group showed a persistent high abundance of *Veillonella* across all seven days. Other taxa, such as *Paenibacillus*, were notably abundant on both the Day1 and Day 7. These findings highlight the differential effects of corticosteroid treatment on microbial dynamics, with the untreated group exhibiting greater diversity over time.

***Differentially Abundant Taxa for Steroid (+) Patients vs. Steroid (-) Patients Across Multiple Time Points***

Differential abundant microbial communities were associated with corticosteroid use in SCAP patients, and **Supplementary Figure 4** shows volcano plots comparing Steroid (+) and Steroid (-) groups.

In the Steroid (+) group, temporal shifts in microbial taxa were evident. From Day 1 to Day 3, taxa such as Phascolarctobacterium and Belnapia showed increased abundance, suggesting that short-term corticosteroid use may stimulate specific microbial populations. However, by Day 7, multiple taxa, including Comamonas, exhibited significant downregulation, indicating a suppressive effect of prolonged corticosteroid use.

***Trends in Relative Abundance of Microbial Genera within Seven Days***

Dynamic changes in microbial genera over seven days were analyzed to explore the effects of corticosteroid treatment. Using average abundance data and K-means clustering, we compared trends between corticosteroid-treated and untreated groups (**Supplementary Figure 5**).

In the Steroid (+) group, *Klebsiella* showed an initially increase followed by a slight decline, while it steadily increased in the untreated group. *Streptococcus* showed a decreasing trend in the Steroid (+) group, whereas its abundance initially declined and then stabilized in the Steroid (-) group. *Pseudomonas* remained stable initially in the Steroid (+) group but increased later, contrast with continuous rise in the Steroid (-) group. These findings suggested corticosteroid treatment may suppress the growth of *Klebsiella* and *Streptococcus* while promoting an increase in *Pseudomonas*.

**Supplementary Tables**

**Supplementary Table 1.** Comparison of traditional bacterial culture methods (TBC) and 16S rRNA sequencing (NGS).

| **Bacterial Species** | **TBC** | **NGS(n)** | **TBC/NGS** |
| --- | --- | --- | --- |
| *Achromobacter* | 1 | 17 | 0.0588 |
| *Acinetobacter* | 21 | 452 | 0.0465 |
| *Burkholderia* | 5 | 131 | 0.0382 |
| *Citrobacter* | 3 | 36 | 0.0833 |
| *Escherichia* | 10 | 399 | 0.0251 |
| *Elizabethkingia* | 2 | 95 | 0.0211 |
| *Enterobacter* | 1 | 10 | 0.1000 |
| *Haemophilus* | 3 | 243 | 0.0123 |
| *Klebsiella* | 35 | 429 | 0.0816 |
| *Moraxella* | 2 | 68 | 0.0294 |
| *Proteus* | 2 | 28 | 0.0714 |
| *Providencia* | 5 | 19 | 0.2632 |
| *Pseudomonas* | 66 | 458 | 0.1441 |
| *Ralstonia* | 9 | 306 | 0.0294 |
| *Serratia* | 4 | 89 | 0.0449 |
| *Staphylococcus* | 22 | 447 | 0.0492 |
| *Stenotrophomonas* | 23 | 298 | 0.0772 |
| *Streptococcus* | 1 | 536 | 0.0019 |

**Supplementary Table 2.** Comparison of traditional culture and NGS in Day 1.

| **Bacterial Species** | **NGS** | **Both** | **Culture** |
| --- | --- | --- | --- |
| *Achromobacter* | 2 | 0 | 0 |
| *Acinetobacter* | 108 | 1 | 0 |
| *Burkholderia* | 29 | 1 | 0 |
| *Citrobacter* | 8 | 0 | 1 |
| *Elizabethkingia* | 16 | 2 | 0 |
| *Enterobacter* | 1 | 0 | 3 |
| *Escherichia* | 121 | 7 | 0 |
| *Haemophilus* | 72 | 2 | 0 |
| *Klebsiella* | 106 | 16 | 0 |
| *Moraxella* | 4 | 2 | 0 |
| *Parvimonas* | 43 | 0 | 0 |
| *Proteus* | 8 | 2 | 0 |
| *Providencia* | 4 | 1 | 0 |
| *Pseudomonas* | 97 | 23 | 1 |
| *Ralstonia* | 75 | 1 | 0 |
| *Serratia* | 23 | 2 | 0 |
| *Staphylococcus* | 115 | 9 | 2 |
| *Stenotrophomonas* | 58 | 1 | 0 |
| *Streptococcus* | 181 | 1 | 0 |
| *Veillonella* | 137 | 0 | 0 |

**Supplementary Table 3.** Comparison of traditional culture and NGS in Day 3.

| **Bacterial Species** | **NGS** | **Both** | **Culture** |
| --- | --- | --- | --- |
| *Achromobacter* | 2 | 0 | 1 |
| *Acinetobacter* | 105 | 8 | 1 |
| *Burkholderia* | 21 | 2 | 0 |
| *Citrobacter* | 9 | 1 | 0 |
| *Elizabethkingia* | 14 | 0 | 0 |
| *Enterobacter* | 6 | 1 | 1 |
| *Escherichia* | 109 | 2 | 0 |
| *Haemophilus* | 45 | 1 | 0 |
| *Klebsiella* | 114 | 7 | 0 |
| *Moraxella* | 9 | 0 | 0 |
| *Parvimonas* | 43 | 0 | 0 |
| *Proteus* | 6 | 0 | 0 |
| *Providencia* | 5 | 2 | 0 |
| *Pseudomonas* | 105 | 17 | 1 |
| *Ralstonia* | 59 | 4 | 0 |
| *Serratia* | 26 | 1 | 0 |
| *Staphylococcus* | 114 | 7 | 0 |
| *Stenotrophomonas* | 58 | 4 | 1 |
| *Streptococcus* | 166 | 0 | 0 |
| *Veillonella* | 122 | 0 | 0 |

**Supplementary Table 4.** Comparison of traditional culture and NGS in Day 7.

| **Bacterial Species** | **NGS** | **Both** | **Culture** |
| --- | --- | --- | --- |
| *Achromobacter* | 2 | 0 | 0 |
| *Acinetobacter* | 111 | 11 | 0 |
| *Burkholderia* | 23 | 2 | 0 |
| *Citrobacter* | 14 | 1 | 0 |
| *Elizabethkingia* | 26 | 0 | 0 |
| *Enterobacter* | 0 | 0 | 2 |
| *Escherichia* | 77 | 1 | 0 |
| *Haemophilus* | 35 | 0 | 0 |
| *Klebsiella* | 103 | 12 | 0 |
| *Moraxella* | 4 | 0 | 0 |
| *Parvimonas* | 36 | 0 | 0 |
| *Proteus* | 6 | 0 | 0 |
| *Providencia* | 1 | 2 | 0 |
| *Pseudomonas* | 89 | 24 | 0 |
| *Ralstonia* | 58 | 3 | 1 |
| *Serratia* | 19 | 1 | 0 |
| *Staphylococcus* | 108 | 4 | 0 |
| *Stenotrophomonas* | 64 | 17 | 0 |
| *Streptococcus* | 140 | 0 | 0 |
| *Veillonella* | 94 | 0 | 0 |

**Supplementary Figures**


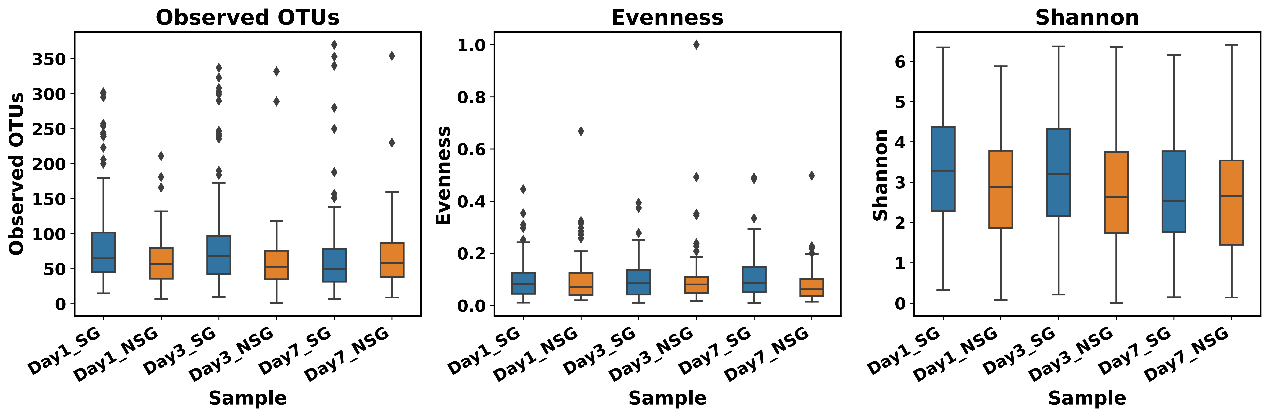


**Supplementary** **Figure 1. Alpha Diversity Analysis by Day and Corticosteroid Treatment Status.** A general observation showed that on both the first and third days, patients treated with corticosteroids exhibited slightly lower OTU counts and Shannon index values compared to untreated patients. However, these observations primarily suggest trends without statistical significance. SG: Steroid (+) group, patients treated with corticosteroids; NSG: Steroid (-) group, untreated patients.


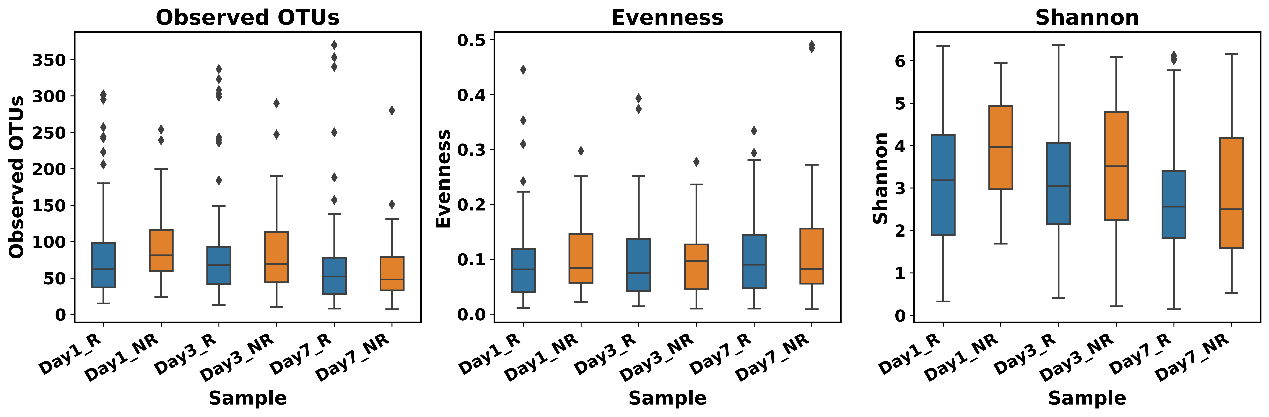


**Supplementary** **Figure 2. Alpha Diversity Analysis by Day and Corticosteroid Responder Status.** A general observation showed that on both the first and third days, responders exhibited slightly lower OTU counts and Shannon index values compared to non-responders. These observations primarily suggest trends without statistical significance. R: Responder group, survivors; NR: Non-Responder group, non-survivors.

**
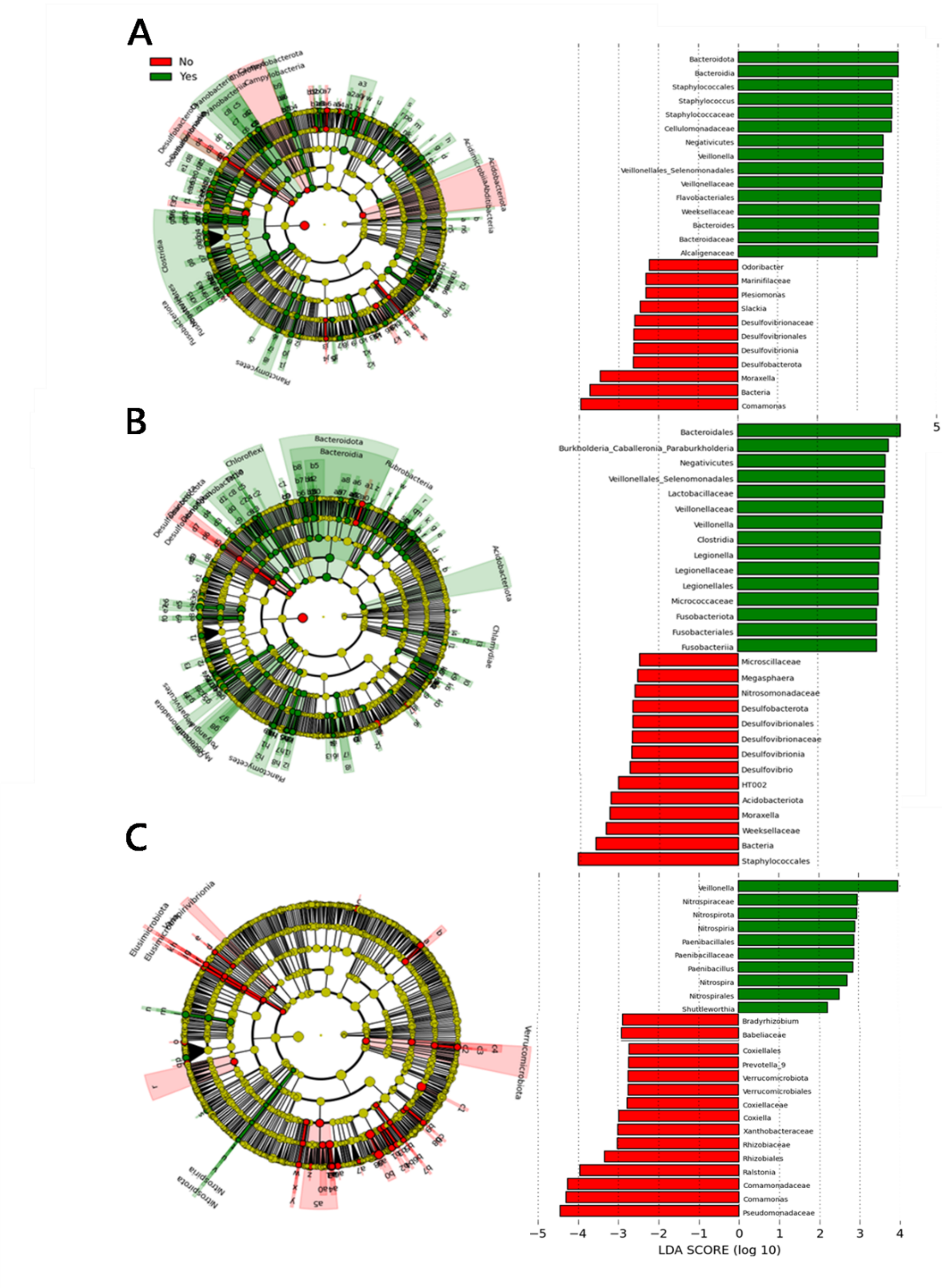
**

**Supplementary Figure 3.** **LEfSe analysis of microbial trends in patients with and without corticosteroid treatment.** Panels A, B, and C represent analysis results from Day 1, Day 3, and Day 7, respectively. Bar charts show the top 15 differentially abundant species, with red bars indicating higher abundance in patients without corticosteroid treatment and green bars indicating higher abundance in patients with corticosteroid treatment.


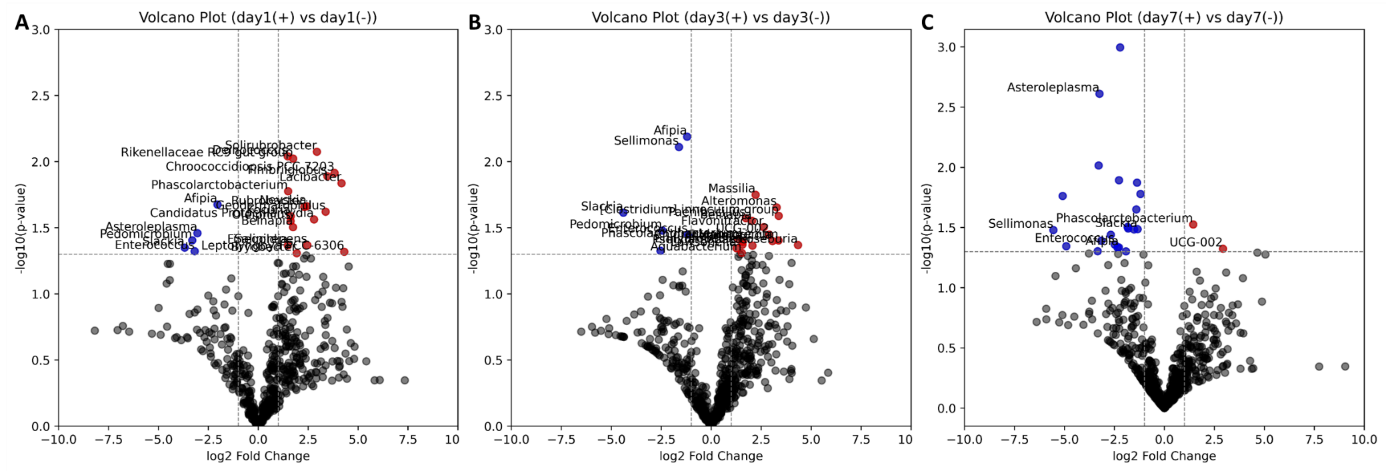


**Supplementary Figure 4. Volcano plots illustrating microbial differences by corticosteroid treatment.** Panels A, B, and C represent analysis results from Day 1, Day 3, and Day 7, respectively. Red dots represent species with log_2_(FC) > 1 and p-value < 0.05, while blue dots represent species with log_2_(FC) < -1 and p-value < 0.05.

**
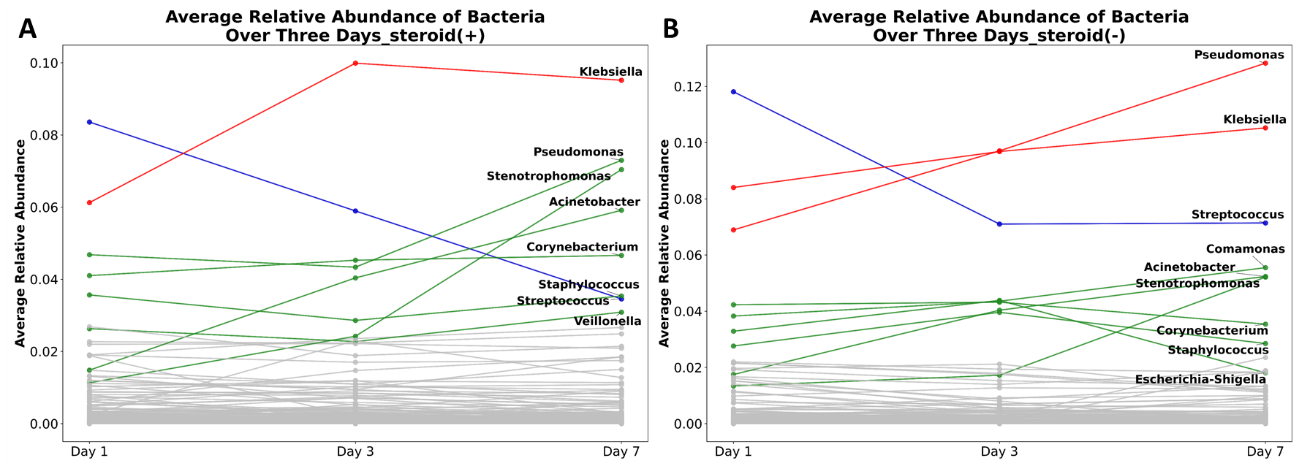
**

**Supplementary Figure 5.** **Temporal trends in relative abundance of microbial genera over seven days.** Panels A and B represent trends of microbial abundance from Day 1 to Day 7 for Steroid (+) and Steroid (-), respectively.
